# COVID-19 mRNA Vaccination Mimicking Heart Attack in a Healthy 56-Year-Old Physician

**DOI:** 10.3390/idr14010011

**Published:** 2022-01-27

**Authors:** Ioannis Xinias, Antigoni Mavroudi, Georgios-Theofilos Theodorou, Ioannis Roilidis

**Affiliations:** 1 Third Pediatric Department, Hippokration Hospital, 54642 Thessaloniki, Greece; xinias@email.com (A.M.); iroilidis@gmail.com (I.R.); 2 Laboratory of Clinical Neurophysiology, AHEPA University Hospital, Aristotle University of Thessaloniki, 54621 Thessaloniki, Greece; theofilos.th@gmail.com

**Keywords:** COVID-19 mRNA vaccine, side-effects, angina

## Abstract

We report our experience regarding a 56-year-old physician who developed severe symptoms mimicking a heart attack a few days after receiving the second dose of the new mRNA vaccine of Pfizer-BioNTech for COVID-19 protection. The patient is a healthy individual with no comorbidities and a normal clinical and laboratory profile. Five days after receiving the second dose on his left shoulder, he manifested sudden, severe pain on the whole left arm which lasted for about one hour, gradually intensifying and migrating to the chest and presenting as severe angina or heart attack. All work-up, however, was negative, with no evidence of ischemic heart attack or myocarditis. Severe acute symptoms lasted for about 20 h and completely resolved after 36 h. Although myocarditis as a potential side effect of the vaccine with associated heart pain has been identified, chest pain mimicking heart attack with otherwise normal workup has not been reported. Physicians must consider this likely rare and self-resolving symptom in order to increase awareness and prevent themselves and their patients from increased anxiety and unnecessary laboratory investigations.

## 1. Introduction

At the end of 2019, a new coronavirus was detected in a group of patients in China after an outspread of an unfamiliar type of pneumonia. Very soon, the virus was found to be transmittable very easily from person to person, and the WHO declared it a pandemic in 2020. Today, it seems that the absolute preventing measure could be active vaccination against the virus [1,2]. Within 12 months from the discovery and publication of the RNA sequence of SARS-COV2, two mRNA vaccines against COVID-19 have been licensed and are broadly used in many relevant parts of the world. The development of this innovative vaccine technology has become of strategic relevance in the best sense of “pandemic preparedness” not only now but also for the future.

Allergic and other reactions against the vaccine and its components have been reported but are still considered uncommon, classifying the vaccines as safe; however, on-going documentation of possible links with side effects and other immune phenomena is important. In case of allergic reactions against vaccine components such as polyethylene glycol (PEG), it is possible to prove the relation with the vaccine by using skin-prick tests [3]. However, with other side effects, more systematic such as angina which could precipitate other acute health conditions, such as a heart attack or myocarditis, one can only speculate on the link via exclusion of these other conditions. This, however, usually remains a speculation alone with no objective proof, and it is only via mass reporting that such side effects can be associated with vaccinations over a long period of time to prove the longstanding safety of the new genetic technology vaccines.

A review of the literature certainly shows a plethora of similar cardiovascular adverse events. These, however, are considered rare and emphasis is given on the efficacy and safety of the new technology mRNA vaccines and the benefits against the COVID-19 pandemic, which outweigh these potential rare side effects [4]. Such rare cardiovascular events that have been reported include myocarditis [4,5], pericarditis, paroxysmal ventricular arrythmia, tachycardia, bradycardia [5,6], myocardial infarction, hypertension, and Takotsubo cardiomyopathy [5]. These adverse events seem to be associated in a timely manner with the vaccine use, but their etiologic relationship cannot be proven yet [5]. Additionally, SARS-CoV-2 infection remains a more serious risk factor for pericarditis, myocarditis, arrythmia, myocardial infarction, and pulmonary embolism then the risk posed by vaccination [4]. Only myocarditis so far has clearly been associated with the mRNA vaccines, and specific epidemiological characteristics have been described. It is a rare adverse effect, and the majority of the cases occur after the second dose of the mRNA vaccine [7]. There is a male predominance and mostly young men under 30 years of age are affected [7,8]. To our knowledge, angina as a separate symptom has not previously been mentioned in the literature.

## 2. Case Presentation

The reported case is that of a healthy 56-year-old male healthcare worker in our hospital who was vaccinated against COVID-19 with a new mRNA vaccine in compliance with the latest national guidelines on vaccine mandates in healthcare workers.

The first dose was administered at the vaccination center of our hospital with no serious side effects. Only mild muscle pain was noted at the injection site, at the left shoulder, which resolved after 72 to 96 h.

Three weeks after the first dose, the patient received the second dose of the same vaccine with no acute side effects.

Symptoms started on the second day after vaccination with pain at the same injection site and swelling at the left axilla, which again resolved after a few days. However, on the fifth day after vaccination, the patient was awoken in the early morning hours by a sharp, strong pain of his whole left upper limb. The patient received 100 mg oral nimesulide and 500 mg oral acetylsalicylic acid. The limb pain resolved after an hour but migrated with increased intensity to the chest, mimicking angina of a heart attack. Due to the persistence of the symptom and worry about a heart attack due to positive family history, he received another 500 mg oral acetylsalicylic acid. After approximately 6 h of persistent severe angina and increasing anxiety, he presented to hospital.

**Figure 1:**
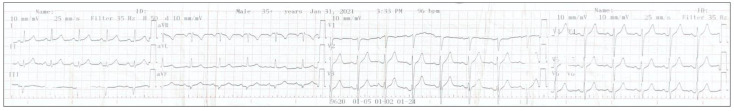
ECG of the patient showing normal pattern.

**Figure 2:**
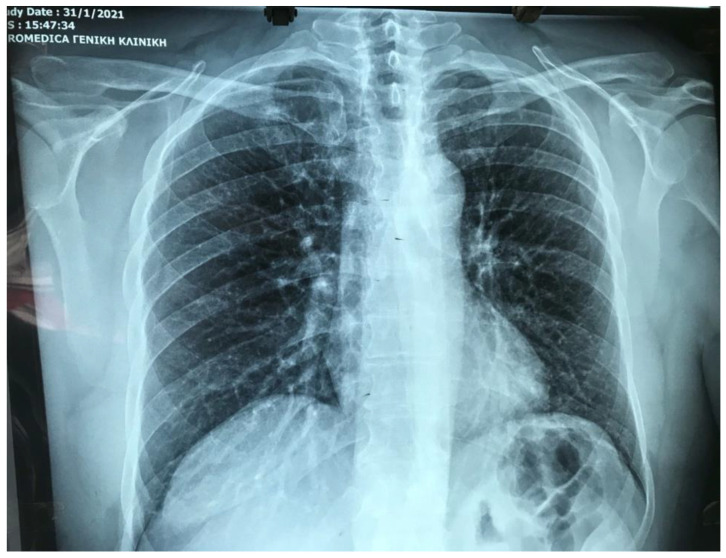
Chest radiography of the patient showing normal radiological findings.

On presentation, the angina was the only symptom with no associated fever, respiratory, or gastrointestinal signs. A full clinical and laboratory examination was undertaken to exclude a heart attack because of the presenting symptoms and family history of coronary artery heart disease. Initial laboratory findings, including blood tests, were not indicative of any heart disease (Table 1), whilst ECG and chest radiography were unremarkable (Figure 1 and Figure 2). After 3 h stay in the hospital’s emergency department, despite the chest pain still ongoing, the patient was reassured of no heart disease or other pathological condition and was discharged home. Further analgesics and rest also did not result in any immediate relief, and only after 24 h since first symptom onset did the patient start feeling gradual resolution of the level of pain, which completely resolved after 36 h.

## 3. Discussion

Within 12 months since the RNA sequence of SARS-COV2 was published, two RNA vaccines against COVID-19 have been licensed and are broadly used in many parts of the world [9,10]. One of them was developed from the company Pfizer-BioNTech and was licensed for use in many countries worldwide. Nowadays, millions of people have been administered this specific vaccine, with side effects reported and now included in the drugs Summaries of Product Characteristics (SPC). However, due to fast-track development of this new vaccine, many side effects would not have been included in the SPC, and the reporting of side effects by physicians is imperative. All the available data on COVID-19 vaccine side effects have been published by manufacturer-funded studies, which are of course in compliance with the Food and Drug Authorities’ guidelines and monitored by third parties [11]. This lack of independent studies on vaccines safety may adversely impact the vaccine uptake and thus slow down vaccine roll-out programs, which are imperative to be accelerated in the next few months [12].

All available evidence so far supports the conclusion that mRNA vaccines are beneficial but may cause some adverse effects including pain, redness, or swelling at the site of the vaccine shot, nausea, vomiting, fever, fatigue, headache, muscle pain, itching, chills, and joint pain, as well as, in rare cases, an anaphylactic reaction [13]. In a recent study among healthcare workers, symptoms following vaccination from the cardiovascular system included heart palpitations (4.36%, 35/803), chest pain (1.12%, 9/803), blood pressure changes, and syncope (0.87%, 7/803) [14]. In the same study, musculoskeletal symptoms which were reported by recipients included myalgia (45.7%, 367/803), arthritis or joint pain (16.6%, 133/803), and muscle stiffness and/or spasm (9.6%, 77/803).

From available data so far, there have been no cases reported of mimicking heart attack symptoms after vaccination with the new mRNA vaccine [13,14,15]. This could be due to under-reporting since, as in our case, many of these are late adverse effects that are not communicated to the primary physician. In addition, since vaccinations are carried out in public vaccination units with only a short post-vaccination monitoring period and not in a family doctor practice setting, closer follow-up of possible late adverse reactions becomes more unlikely.

Moreover, in regards to the observed rates of side effects in clinical trials, these do not always reflect those observed in clinical practice. This is due to the fact that clinical trials are conducted under widely varying conditions and adverse reaction rates observed are not always comparable to those observed in another clinical trial of another vaccine [13].

## 4. Conclusions

Physicians must be aware of this unlikely, however possible, late onset and previously unreported side effect of the new mRNA COVID-19 vaccine. Awareness will allow them to inform their patients and potentially avoid anxiety and unnecessary investigations.

As a preventative measure healthcare personnel who vaccinate may prefer to do so in the right arm so that, in case such a side effect occurs, it may be more localized in the right, thus making it less likely to be associated with a cardiac problem.

In addition, a general practitioners practice when assessing patients who present shortly after vaccination with pain that could mimic a heart attack should take into consideration the age, cardiac health history, time from vaccination and onset of symptoms, and site of vaccination, as mentioned above, in conjunction with an ECG as a minimum test in order to stratify how much of an emergency this might be.

## Figures and Tables

**Table 1:** Laboratory findings of the patient at the acute phase of the symptoms (at 6 h from the on-set).

Test	Result	Normal Levels
Troponine (pg/mL)	2,1	<34,2
CK MB (U/L)	16	<25
WBC (/μL)	10,260 (PMN: 77,3%)	4000–10,000
Triglycerides (mg/dL)	63	<150
CRP (mg/dL)	0,91	<0,3
LDL (mg/dL)	118	<115
HDL (mg/dL)	69	32–68
Urea (mg/dL)	29	10–50
Creatinine (mg/dL)	1,02	0,70–1,30
Glucose (mg/dL)	110	70–110
